# Plastic Mulching Film and Straw Return Alter Starch Physicochemical and Tuber Textural Properties of Intercropping Potatoes

**DOI:** 10.3390/foods14183179

**Published:** 2025-09-12

**Authors:** Zhenpeng Deng, Guangyan Sun, Keyou Zhou, Mingcong Li, Fengming Liang, Jichun Wang, Changwen Lyu

**Affiliations:** 1 College of Agronomy and Biotechnology, Southwest University, Chongqing 400715, China; zhenpenger@gmail.com (Z.D.); sunguangyan98@163.com (G.S.); 2 Key Laboratory of Biology and Genetic Breeding for Tuber and Root Crops in Chongqing, Chongqing 400715, China; 3 Agricultural Technology Extension Center of Wuxi, Chongqing 405800, China; 4 Wuxi Shuguang Agricultural Technology Development Company, Chongqing 405800, China

**Keywords:** potato, plastic mulching, straw returning, intercropping, starch physicochemical properties, textural properties

## Abstract

To analyze the impact of intercropping, maize straw returning, plastic mulching, and different configurations on potato quality in southwest China, a three-factor split-plot field experiment was designed to investigate the effects of crop management practices on the starch physicochemical properties and textural properties for two potato cultivars (Mira and Huayu-5). Results indicated that intercropping, maize straw returning, and plastic mulching reduced tuber dry matter, total starch, and amylose content, thereby decreasing the hardness of steamed tubers. Plastic mulching and maize straw returning increased starch granule size, promoted thermal properties, improved pasting properties, and increased adhesiveness and cohesiveness. The potato/maize relay intercropping increased the thermal properties, pasting viscosities, adhesiveness, and cohesiveness, with stronger effects observed in Mira compared to Huayu-5. The combination of intercropping with plastic film mulching and straw returning reduces tuber hardness while enhancing tuber adhesiveness and cohesiveness.

## 1. Introduction

Potato (*Solanum tuberosum* L.) is one of the most important crops and ranks as the fourth most cultivated crop globally. Over one billion people rely on potato tubers as a premium staple food due to the various healthy and versatile nutritious components that include carbohydrates, protein, vitamin C, vitamin B6, magnesium, potassium, and dietary fiber [1]. Also, potato is cultivated as a vegetable for consumption and a raw material for processing into products, such as starch, potato chips, and French fries.

**Figure 1:**
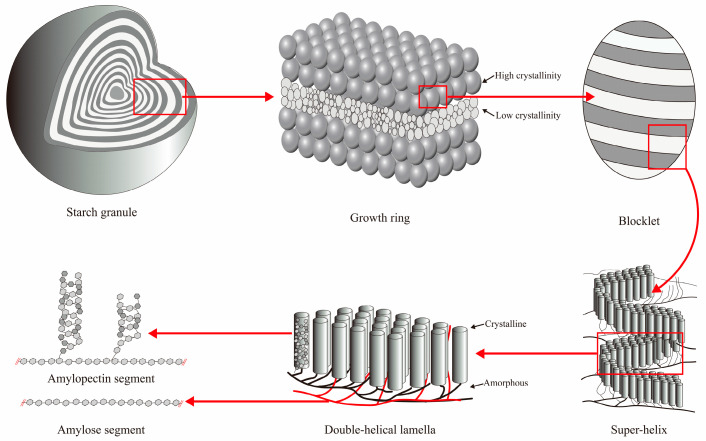
The multi-level structure of the starch granule.

Starch is a fundamental component of the nutritious components in potato tubers, accounting for approximately 70–85% of dry weight, and consists of amylopectin and amylose forming semi-crystalline starch granules [2]. The starch granule is a semi-crystalline multi-level entity with specific structural features (Figure 1). The starch granule is characterized at the molecular level mainly by the essentially linear α-1,4 glucan amylose and the branched α-1,4; α-1,6 glucan amylopectin, at the 8–11 nm level by crystalline and amorphous lamellar, and at the 0.1 μm scale by alternating amorphous and semi-crystalline growth rings [3]. It is noteworthy that different-sized starch granules show varied molecular components and physicochemical properties [4,5]. For potatoes, smaller granule size was associated with lower amylose content [6]. It is known that differences in the composition, architecture, and dimensions of starch granules have impacts on their functional performance in foods, such as swelling power, gelatinization, retrogradation, flowability, digestibility, and textural properties [7,8]. Amylose always has a positive correlation with harder texture. Amylose molecules can entangle with amylopectin chains and form co-crystals within the crystalline lamellae, thereby restricting starch granule swelling [9]. The starch FTIR-ATR spectrophotometry was used to investigate the short-range order of starch granules. The IR bands at 1045, 1022, and 995 cm^−1^ are associated with crystalline regions, amorphous regions, and bonding in hydrated carbohydrate helices, respectively, while the 1045/1022 cm^−1^ ratio (IR1045/1022) was calculated to estimate the degree of ordered structures and the 1022/995 cm^−1^ ratio (IR1022/995) was used to determine the amorphous-to-ordered structure index in the external regions of starches [10]. Additionally, methods such as the Rapid Visco Analyser (RVA) profiles, differential scanning calorimetry (DSC) curves, and texture analysis can be employed to assist in evaluating the eating quality. The RVA parameters, such as peak viscosity, breakdown viscosity, setback viscosity, and gelatinization temperature, have shown strong correlations with eating quality. Generally, higher peak viscosity indicates a softer texture, while a lower setback viscosity corresponds to a reduced tendency of starch retrogradation after cooling. The physicochemical properties of potato starch affect the sensory characteristic of texture.

The architecture and composition of the native starch granule depend on the genotype, but also on the crop management practices [11,12,13]. Plastic mulching film (PMF) is one of the key cultivation practices to improve potato yields by altering soil temperature, moisture retention, and weed inhibition [14]. The PMF treatment increases starch content and improves the quality of sweet potato [15]. In addition, potato/maize relay intercropping (MIP), as a cereal–solanaceae intercropping system, is widely practiced in many regions of the world. Potato intercropping with maize can reduce photosynthetically active radiation and micro-environment temperatures, leading to changes in the starch structure and textural properties. The shading by the higher crop (maize) modifies the light environment experienced by the lower crop (potato) in terms of both light quantity and quality [16]. Low-light stress reduces amylose content and diminishes the proportion of large starch granules, thereby enhancing swelling capacity, improving water solubility index, and increasing gelatinization enthalpy [17]. Post-harvest maize stalks can be used as organic fertilizer to improve potato yield and quality by enhancing soil organic matter and microbial activity, and increasing essential nutrients [11]. For example, straw returning (SRT) can improve rice amylose content and protein content, which alters the rice cooking and eating quality [18]. However, to the best of our knowledge, the effects of management practices (plastic mulching film, straw returning, intercropping with maize) on the multi-level structures and functional properties of potato starch have not been studied.

Intercropping, straw returning, and mulching are important agronomic practices for potatoes and have a significant impact on yield. Cultivation practices can change the environment in which potatoes are grown, and these environmental changes can, in turn, affect the composition and physicochemical properties of potato starch. However, the specific effects of cultivation practices on potato starch composition and properties are still being investigated. Therefore, we hypothesize that different cultivation practices will lead to changes in starch composition and granule size, which in turn will lead to changes in starch thermal properties and pasting viscosities, altering starch water solubility and swelling power and ultimately affecting the texture of steamed tubers. A better understanding of the relationships between starch components, starch multi-level structures, starch physicochemical properties, and textural properties of potatoes under different management practices may help to provide a theoretical basis for the optimum management practices of better-quality potatoes and improve starch processing and eating quality in potatoes.

## 2. Materials and Methods

### 2.1. Plant Materials and Field Experiment

**Figure 2:**
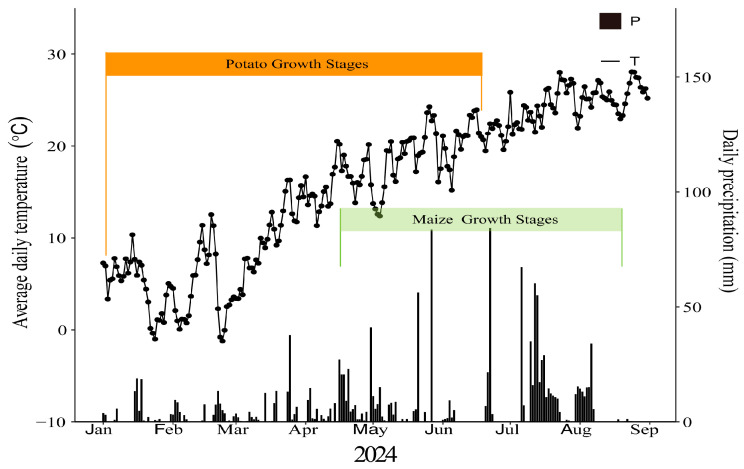
Mean daily air temperature (lines and points) and precipitation (bars) across study site-years.

**Figure 3:**
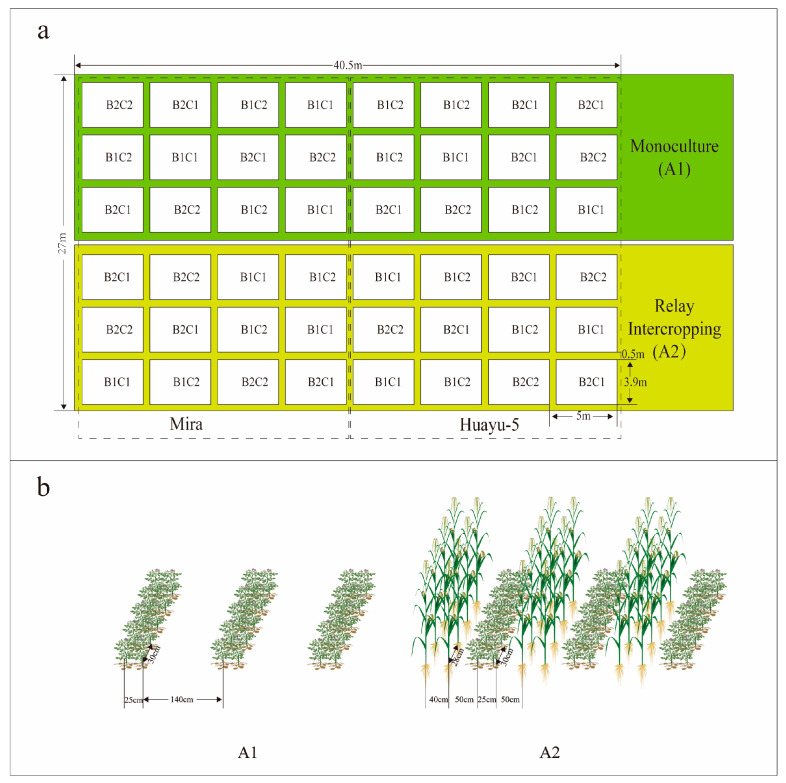
Experimental plot layout (**a**) and schematic diagram of cropping systems (**b**). A1: potato monoculture; A2: the potato/maize relay intercropping system. B1: without maize straw; B2: maize straw returning. C1: without mulching; C2: plastic film mulching.

Two potato cultivars that are widely used in southwest China, Huayu-5 and Mira, provided by the Key Laboratory of Biology and Genetic Improvement for Tuber and Root Crops of Chongqing, were grown in 2024 at a research site in Wuxi, Chongqing, China (109.7° E, 38.3° N). Huayu-5 is an early–medium maturing variety with a growing period of 78 days, while Mira is a medium–late maturing variety with a growing period of 105 days. Meteorological data can be found in Figure 2. The characteristics of the 0–20 cm soil layer were as follows: pH value of 6.67, 27.54 g/kg organic matter, 0.89 mg/g total soil nitrogen, 1.34 mg/g total soil phosphorus, 18.01 mg/g total soil potassium, 11.49 mg/kg available nitrogen, 11.49 mg/kg available phosphorus, and 68.85 mg/kg available potassium. The field experiment was laid out as a randomized complete block design and performed in triplicate. A three-factor split-plot design was used for this experiment, with factor A consisting of two cropping systems (A1: potato monoculture; A2: the potato/maize relay intercropping system), factor B of two straw returning levels (B1, without maize straw; B2, maize straw returning), and factor C of two mulching models (C1, without mulching; C2, plastic film mulching). The plot area was 19.5 m^2^ (3.9 m × 5 m). For A2, the whole intercropping strip width was 1.67 m, the row spacing between maize and potato rows was 0.37 m and 0.25 m, and the distance between adjacent maize and potato rows was 0.5 m. For potato monoculture, the distance between two rows was 1.42 m (Figure 3). Potatoes were planted on 10 January and harvested on 14 June. Maize seeds were sown in the wet nursery on 21 March, and seedlings were transplanted to the field on 9 April and harvested on 23 August. The maize straw was cut into lengths of approximately 10 cm and decomposed for one month, and 60 kg of phosphate fertilizer and 200 kg of manure were added to every 500 kg of dry straw. The amount of decomposed straw returned to the field was 22,500 kg per hectare. A total of 1125 kg of compound fertilizer (N-P_2_O5-K_2_O is 15-10-15) was applied per hectare, and was applied as base fertilizer during sowing. The black plastic mulch had a thickness of 0.008 mm and a width of 1 m. Manual weeding was conducted, and diseases were controlled via several applications of chemicals from planting to harvest to avoid yield losses. No irrigation was applied during the whole reproductive period. Other field management followed standard practices that were advised by the local agricultural technology extension center.

### 2.2. Dry Matter and Starch Isolation

Uniform-sized potatoes were selected from each treatment before starch isolation. The tubers were peeled, sliced, and grounded at high speed in a blender with distilled water (1:1 *v*/*v*) at 25 °C for 3 min. The homogenate was passed through sieves (0.250 and 0.180 mm screens), and the solids retained were washed three times on the sieve. The filtrate was left to stand overnight at 5 °C for decantation. The starch sediment was washed with distilled water and ethanol, and dried at 38 °C in an air circulation oven. The dry weight of each sample was determined after drying for 72 h at 85 °C in a forced-air drying oven. The samples were then weighed to calculate dry matter, and ground into a fine powder to measure the starch composition.

### 2.3. Starch Composition and Granule Size Analysis

The starch samples were suspended in water, and then a laser-diffraction particle-size analyzer (Mastersizer 3000, Malvern, England) was used to measure particle-size distribution [19]. The total starch, apparent amylose, and amylopectin contents of dry tuber samples were determined by colorimetric method according to Ahmed et al. [13].

### 2.4. Determination of Starch Pasting Properties and Thermal Properties

The pasting behavior of starches was measured with a Micro Visco-Amylo-Graph (MVAG, Brabender, Duisburg, Germany) through the method of Waleed et al. [20]. The main viscosity parameters were obtained from the pasting curves. The gelatinization properties of starches were studied following the modified method of Zhang et al. [21]. Starch (3 mg) and distilled water (6 μL) were added into an aluminum pan and kept in a refrigerator at 4 °C for 12 h. Then, the samples were heated from 30 °C to 100 °C at a heating rate of 10 °C/min using a differential scanning calorimetry (PerkinElmer, DSC4000, USA) that was equipped in Beibei District, Chongqing, China.

### 2.5. Starch FTIR-ATR Spectrophotometry

The short-range ordered structures of starches were analyzed using a Fourier transform infrared spectrometer (PerkinElmer, Spectrum Two, Waltham, MA, USA) following the method of Guo et al. [22]. The spectra were set from 400 cm^−1^ to 4000 cm^−1^ with a resolution of 4 cm^−1^ [23].

### 2.6. Determination of Starch Water Solubility and Swelling Power

Water solubility (WS) and swelling power (SP) were determined following the method of Liu et al. [24] with some modifications. In total, 0.3 g starch (m1) and 10 mL distilled water were mixed and transferred to centrifuge tubes, and heated from 50 °C to 90 °C for 30 min in a water bath. Then, the samples were cooled to room temperature and centrifuged (3000 rpm, 15 min). The supernatant was poured into a beaker and dried to a stable weight (m2) at 105 °C, and the remaining starch paste was weighed (m3). The WS and SP were calculated as follows:

WS (%) = (m2/m1) × 100

SP (%) = [m3/(m1 − m2)] × 100

### 2.7. Determination of Textural Properties of Cooked Potatoes

The textural properties of steamed potatoes included hardness, cohesiveness, and adhesiveness. They were evaluated using a texture analyzer (TA. XT Plus990000, Stable Micro System, Godalming, Surrey, UK) that was equipped in Beibei District, Chongqing, China, with a 50 mm cylindrical probe and a two-deformation test (TDT). Potato tubers of uniform size (100–150 g) were selected from each treatment and steamed for 50 min. Then, steamed potato samples were sliced 1 cm thick for the measurements. The parameters used were as follows: the first compression ratio of 20% and the second compression ratio of 80%, a test speed of 5.0 mm/s, a retraction speed of 10.0 mm/s, and a triggering force of 5 g.

### 2.8. Statistical Analysis

Excel 2019 was used for data sorting. The “mvnormtest” in R software (version 4.4.2) was used to perform the Shapiro–Wilk test for normality. The “agricolae” package in R software was used to analyze the significant difference in data by using Tukey’s one-way ANOVA. All values were compared using the least significant difference test (* *p* < 0.05, ** *p* < 0.01, *** *p* < 0.001). Mantel tests were used to gain insights into the correlations by using the “linkET” package in R software. The “ggplot2” package in R software was used to construct graphs.

## 3. Results

### 3.1. Difference in Starch Component and Granule Size Distribution Under Different Crop Management Practices

#### 3.1.1. Dry Matter, Total Starch, Amylose, and Amylopectin Content

Dry matter, total starch, amylose, amylopectin content, and amylose/amylopectin ratio were affected by the potato/maize relay intercropping (A), straw returning (B), and plastic mulching film (C) in Huayu-5 and Mira (Table 1). In Huayu-5, total starch and amylopectin content were significantly affected by A × C interaction. In Mira, dry matter, total starch content, and amylose/amylopectin ratio were significantly affected by A × C and A × B × C interactions. The A2, B2, and C2 treatments decreased tuber dry matter by 16.04%, 9.12%, and 11.79% (Huayu-5) and 11.44%, 3.16%, and 10.23% (Mira), respectively, compared with A1, B1, and C1. In Huayu-5, A2 and C2 decreased total starch content by 10.82% and 13.22%, respectively, compared with A1 and B1 treatments. In Mira, A2 and B2 increased total starch content by 2.79% and 4.97%, respectively, compared with A1 and B1 treatment. Meanwhile, compared with C1, C2 decreased total starch content in Mira by 14.38%. The A2, B2, and C2 treatments decreased amylose content by 13.16%, 11.16%, and 9.01% (Huayu-5) and 9.82%, 10.77%, and 22.46% (Mira), respectively, compared with A1, B1, and C1. The A2, B2, and C2 treatments decreased amylopectin content of amylopectin by 11.68%, 1.56%, and 12.57%, respectively, compared with the A1, B1, and C1 treatments. Compared to C1, C2 decreased amylopectin content in Mira by 11.65%. Meanwhile, compared with B1, B2 decreased amylopectin content in Mira by 6.90%.

#### 3.1.2. Starch Granule Size Distribution

Table 2 indicated that the A treatment had little effect on the medium diameter starch granule (MSG) of Huayu-5, but significantly affected the MSG of Mira. The A1 increased the MSG of Mira by 8.46% to 13.97% compared to A2. The B and C treatments had a significant effect on MSG in Huayu-5 and Mira. The MSG in the treatment of B2 was higher, from 6.64% to 9.15% in Huayu-5 and 1.70% to 7.52% in Mira, than in B1. The MSG in the treatment of C2 was higher, from 4.85% to 13.87% in Huayu-5 and 8.90% to 11.14% in Mira, than in C1. In Mira, the A, B, C treatments and A × B × C interaction had a significant effect on the volume average particle size. In Huayu-5, the volume average particle size was significantly affected by B, C and B × C interaction. C1 increased the volume average particle size by 4.16% in Huayu-5 and 8.28% in Mira compared to C2. The A2 decreased the volume average particle size by 3.34–11.93% in Mira compared to A1. The B and C treatments had a significant effect on specific surface area in both varieties. In Mira, the A2 increased the specific surface area by 4.63–8.48% compared to A1. Compared with C1, C2 decreased the specific surface area by 8.05% (Huayu-5) and 10.56% (Mira), respectively.

#### 3.1.3. ATR-FTIR Spectrophotometry

**Figure 4:**
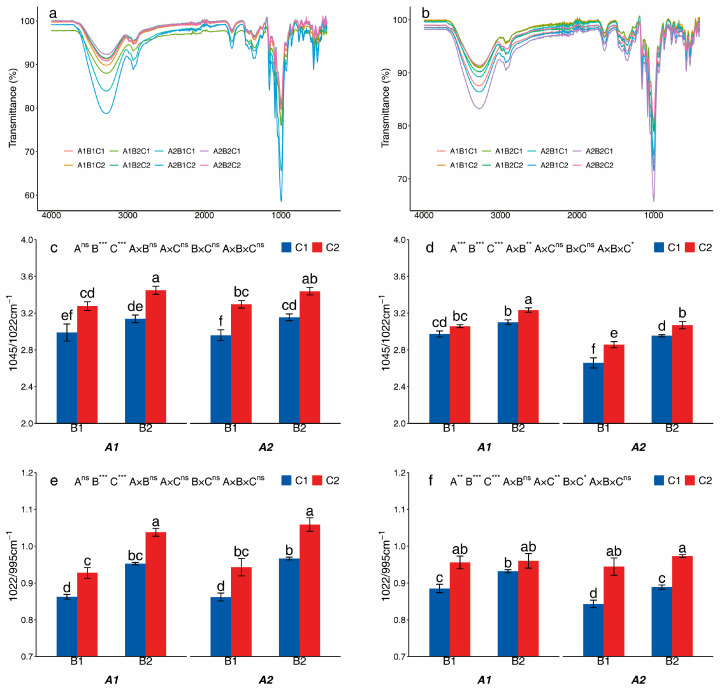
IR Ratios of starches from different treatments in Huayu-5 (**a**,**c**,**e**) and Mira (**b**,**d**,**f**). Different letters in the same figure are significantly different, indicated by Tukey’s one-way at *p* < 0.05. *, significance at *p* < 0.05; **, significance at *p* < 0.01; ***, significance at *p* < 0.001; ns, not significant at *p* > 0.05. A1: potato monoculture; A2: the potato/maize relay intercropping system. B1: without maize straw; B2: maize straw returning. C1: without mulching; C2: plastic film mulching.

The ATR-FTIR spectra of two potato starches in the 4000–900 cm^−1^ region are shown in Figure 4a,b. The ratios for 1045/1022 cm^−1^ (IR1045/1022) and 1022/995 cm^−1^ (IR1022/995) of starches from different treatments are shown in Figure 4c–f. The A treatment had little effect on the IR1045/1022 and IR1022/995 of Huayu-5, but significantly affected the IR1045/1022 and IR1022/995 of Mira. The B and C treatments significantly affected the IR1045/1022 and IR1022/995 in both varieties. In Huayu-5, B2 and C2 treatment increased IR1022/995 by 5.27% and 9.96%, respectively, compared with B1 and C1. Similarly, in Mira, B2 and C2 treatments increased IR1022/995 by 7.04% and 4.55% and increased IR1045/1022 by 3.44% and 7.99% compared with B1 and C1. The A2 treatment decreased IR1045/1022 and IR1022/995 by 7.15% and 2.26%, respectively, compared with A1. The IR1022/995 in Mira was higher in A1B2C2 that was 2.66.

### 3.2. Difference in Starch Physicochemical and Textural Properties Under Different Crop Management Practices

#### 3.2.1. Pasting Properties of Starch

The A treatment did not have an effect on the pasting viscosities in Huayu-5, but significantly affected the pasting viscosities in Mira (Table 3). In Mira, A1 increased the PV by 6.78% to 14.46% compared to A2. The B and C treatments significantly affected the pasting viscosities in both varieties. B2 and C2 increased the PV by 25.19–37.45%, 14.31–25.51% (Huayu-5) and 14.85–27.32%, 9.52–21.42% (Mira), respectively, compared with B1 and C1. B2 and C2 decreased the HV by 13.04–16.99%, 24.01–27.71% in Huayu-5, compared with B1 and C1. In Mira, the C2 increased the HV by 2.80% to 12.37% compared to C1. The B2 and C2 treatments increased the FV by 14.11–24.35% and 5.80–12.57% in Huayu-5, compared with B1 and C1. In Mira, the A2 increased the FV by 1.69% to 11.98% compared to A1. B2 and C2 increased the SB by 33.90–58.27%, 4.72–23.78% (Huayu-5) and 27.95–73.23%, 7.07–44.52% (Mira), respectively, compared with B1 and C1. The BV in Mira was higher in A1B2C2, which was 1774.67 cP, and the lowest was under A2B1C1, which was 757.00 cP.

#### 3.2.2. Thermal Properties

**Figure 5:**
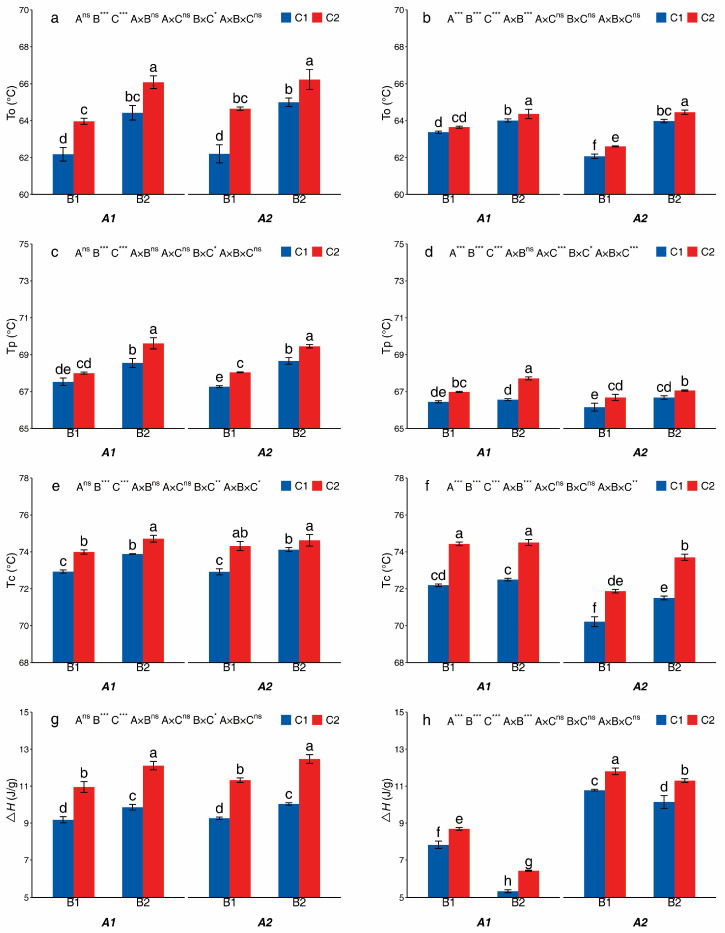
The thermal properties of starches from different treatments in Huayu-5 (**a**,**c**,**e**,**g**) and Mira (**b**,**d**,**f**,**h**). Different letters in the same figure are significantly different, indicated by Tukey’s one-way at *p* < 0.05. *, significance at *p* < 0.05; **, significance at *p* < 0.01; ***, significance at *p* < 0.001; ns, not significant at *p* > 0.05. A1: potato monoculture; A2: the potato/maize relay intercropping system. B1: without maize straw; B2: maize straw returning. C1: without mulching; C2: plastic film mulching.

The A, B, and C treatments had significant effects on the thermal properties of starches in both potato cultivars (Figure 5). The A treatment significantly affected the To, Tp, Tc, and ∆H of Mira. Compared to A1, A2 exhibited decreases in To, Tp, and Tc by 0.90%, 0.43%, and 2.20%, respectively, but an increase in ∆H by 35.98–90.70%. The B and C treatments significantly affected the To, Tp, Tc, and ∆H of both potato cultivars. B2 and C2 increased the To by 2.44–4.49%, 1.89–3.93% (Huayu-5) and 1.00–3.08%, 0.43–0.86% (Mira), respectively, compared with B1 and C1. B2 and C2 increased the Tp by 1.51–2.37%, 0.70–1.55% (Huayu-5) and 0.18–1.09%, 0.81–1.73% (Mira), respectively, compared with B1 and C1. B2 and C2 increased the Tc by 0.42–1.65%, 0.68–1.92% (Huayu-5) and 0.10–2.56%, 2.35–3.08% (Mira), respectively, compared with B1 and C1. Compared to the B1 treatment, the B2 treatment decreased the ∆H by 4.29–32.01% in Mira. Meanwhile, compared with B1, the B2 treatment increased the ∆H by 7.40–10.59% in Huayu-5. The C2 treatment significantly increased the ∆H of Huayu-5 and Mira compared to that of the C1 treatment. In Huayu-5, the ∆H of C2 treatment increased by 19.34% to 24.20% compared to that of the C1 treatment. Similarly, in Mira, the C2 treatment resulted in an increase in the ∆H, ranging from 9.53% to 20.77% compared with the C1 treatment.

#### 3.2.3. Swelling Power and Solubility

**Figure 6:**
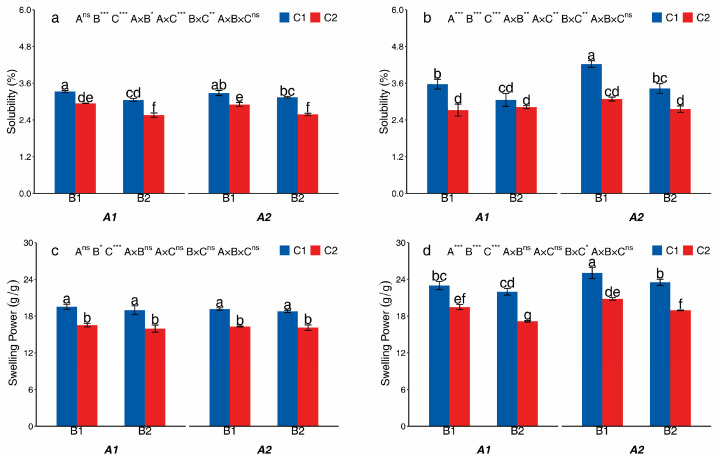
The swelling power and solubility from different treatments in Huayu-5 (**a**,**c**) and Mira (**b**,**d**). Different letters in the same figure are significantly different, indicated by Tukey’s one-way at *p* < 0.05. *, significance at *p* < 0.05; **, significance at *p* < 0.01; ***, significance at *p* < 0.001; ns, not significant at *p* > 0.05. A1: potato monoculture; A2: the potato/maize relay intercropping system. B1: without maize straw; B2: maize straw returning. C1: without mulching; C2: plastic film mulching.

The treatment had a significant effect on the swelling power and solubility of starches in both potato cultivars (Figure 6). In both cultivars, the solubility and swelling power were significantly affected by B and C. The C2 treatment significantly decreased the solubility of Huayu-5 and Mira compared to that of the C1 treatment. In Huayu-5, the solubility of the C2 treatment was reduced by 11.52% to 17.86% compared to that of the C1 treatment. Similarly, in Mira, the C2 treatment resulted in a decrease in the solubility, ranging from 7.82% to 27.06% compared to the C1 treatment. In addition, the C2 treatment significantly decreased the swelling power of Huayu-5 and Mira compared to that of the C1 treatment. The swelling power of Huayu-5 and Mira in the C2 treatment decreased by 14.09–15.80% and 15.30–21.95%, respectively, as compared to the C1 treatment. The A treatment did not significantly affect the solubility and swelling power of Huayu-5. However, in Mira, the A treatment significantly affected the solubility and swelling power. In Mira, the A1 treatment resulted in a 10.92% decrease in the solubility and an 8.27% decrease in the swelling power compared to the A2 treatment.

### 3.3. Textural Properties of Cooked Potatoes

**Figure 7:**
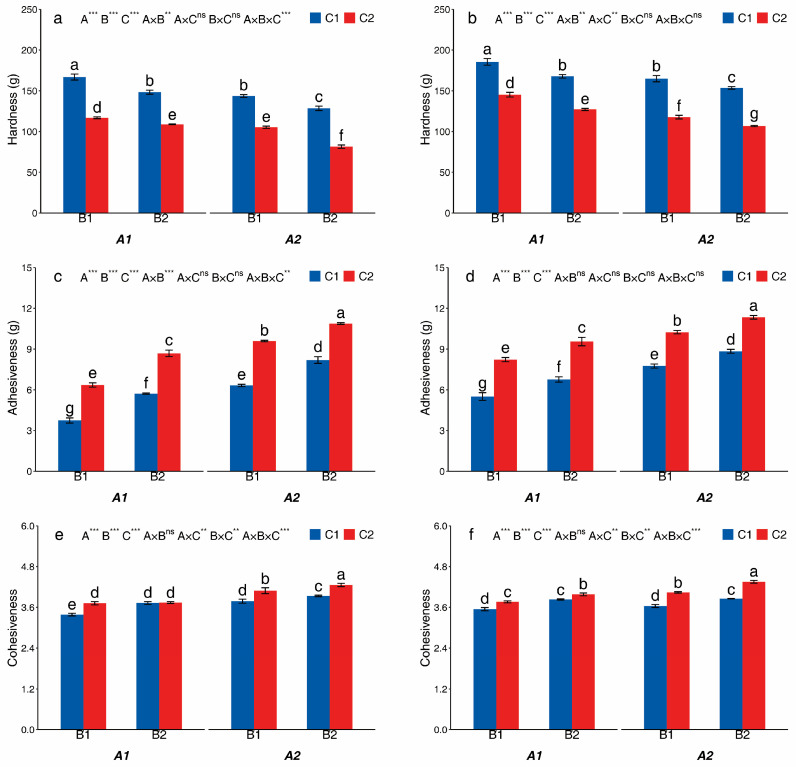
The textural properties of cooked potatoes from different treatments in Huayu-5 (**a**,**c**,**e**) and Mira (**b**,**d**,**f**). Different letters in the same figure are significantly different, indicated by Tukey’s one-way at *p* < 0.05. **, significance at *p* < 0.01; ***, significance at *p* < 0.001; ns, not significance at *p* > 0.05. A1: potato monoculture; A2: the potato/maize relay intercropping system. B1: without maize straw; B2: maize straw returning. C1: without mulching; C2: plastic film mulching.

Textural properties of cooked potatoes were significantly affected by A, B, and C in two cultivars (Figure 7). The A2, B2, and C2 treatments decreased hardness by 9.99–25.13%, 6.97–22.62%, 26.66–36.64% (Huayu-5) and 8.44–18.98%, 6.82–12.45%, 21.73–30.49% (Mira), respectively, compared with A1, B1, and C1. The A2, B2, and C2 treatments increased adhesiveness by 25.27–68.84%, 13.13–52.82%, 32.82–69.92% (Huayu-5) and 18.71–40.88%, 10.71–22.93%, 28.31–49.39% (Mira), respectively, compared with A1, B1, and C1. The cohesiveness of Huayu-5 and Mira in C2 treatment increased by 5.61–13.86% and 0.62–9.40%, respectively, as compared to the C1 treatment. The C2 and B2 treatments increased cohesiveness by 8.79% and 3.93%, respectively, compared with C1 and B1. The tuber cohesiveness was the highest under the A2B2C2 treatment (4.26 for Huayu-5 and 4.30 for Mira) and lowest under the A1B1C1 treatment (3.39 for Huayu-5 and 3.55 for Mira).

### 3.4. Correlations Between Starch Structure and Physicochemical Properties

**Figure 8:**
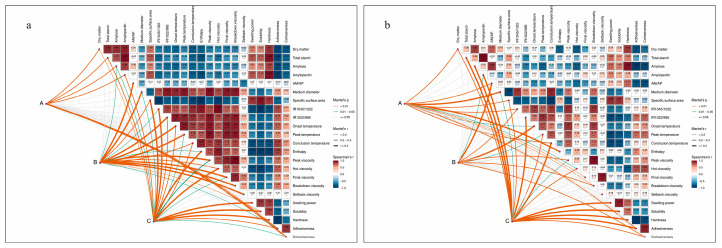
Correlations among the crop management practices, the physiochemical properties of starch, and the textural properties of cooked potatoes in Huayu-5 (**a**) and Mira (**b**). A: cropping systems; B: straw returning levels; C: mulching models. *, significance at *p* < 0.05; **, significance at *p* < 0.01; ***, significance at *p* < 0.001. The line color corresponds to Mantel’s *p* values. The color gradient denotes Pearson’s correlation coefficient. Asterisks indicate the importance (%) of starch to cookie tastes (* *p* < 0.05), as revealed by the random forest model.

The Mantel test was used to determine how A, B, and C affected the composition and physicochemical properties of potato starch (Figure 8). The A was significantly correlated with dry matter, total starch content, amylopectin content, and cohesiveness in Huayu-5, and correlated with dry matter content, amylose/amylopectin ratio, MSG, IR1045/1022, pasting properties (the peak viscosity, hot viscosity, and final viscosity), and adhesiveness in Mira. B was significantly correlated with the amylopectin content, amylose/amylopectin ratio (AM/AP), and thermal and pasting viscosity properties. In both varieties, C deteriorates hardness and adhesiveness by regulating the dry matter content, total starch content, amylopectin content, and hot viscosity. In Mira, the adhesiveness was positively related to the gelatinization temperatures, peak and breakdown viscosity, IR1045/1022, and IR1022/995, while negatively related to dry matter, total starch, and amylose content. In contrast, the hardness was positively related to dry matter, total starch, and amylose content, while negatively related to IR1022/995, enthalpy, and hot viscosity. In Huayu-5, the adhesiveness was positively related to IR1045/1022 and the final, hot, and setback viscosity. The hardness was positively related to dry matter, total starch, and amylose content, while negatively related to IR1045/1022 and IR1022/995, enthalpy, and peak and hot viscosity.

## 4. Discussion

### 4.1. Effects of Crop Management Practices on the Potato Starch Composition and Granule Size

The mulching plastic film (PMF) is an important cultivation factor to improve crop production and affect the composition of starch [25,26]. Previous studies have found that plastic film mulching decreased the tubers’ starch and dry mass contents in comparison to tubers cultivated without any cover [27]. Similar results were reported by Hou et al. [15], who found that the amylose content of sweet potato was decreased under the PMF treatment compared to that under the non-PMF treatment. In this study, the PMF treatment decreased dry matter, total starch content, and amylose content compared with non-PMF. Dry matter and total starch content are affected by several growth environment factors, including temperature, soil moisture, and nutrients [4]. The main reason for the decrease in tuber dry matter, total starch, amylose, and amylopectin content with PMF could be attributed to the temperature rise. PMF can lead to underground temperature increasing [28]. The elevation of temperatures decreased total starch, amylose, and amylopectin content and increased the amylose/amylopectin ratio [29]. The reason may be that high temperatures affect potato growth and development, shortening dry matter accumulation time and inhibiting cell growth and total starch accumulation. Previous studies have shown that the UDPGPPase activity was affected by the PMF treatment [30]. Hence, high temperature reduces the activity of starch biosynthesis enzymes. The lower amylose content in rice grain is caused by high temperature inhibiting the activity of GBSS [31]. Previous studies have shown that the starch content decreased significantly under high temperature stress; this reduction could be due to high temperature exhibiting the expression of genes or the activities of enzymes in starch biosynthesis [32]. In our present study, PMF increased the average diameter of starch granules and enhanced the proportion of large starch granules by volume and surface area, respectively. The number of potato starch granules was increased first at the early filling stage and formed large-sized granules generally at the late filling stage. The high temperature stress in the early stage limits the increase in the number of starch granules, and, at the same time, the nutrients are transferred to the already formed starch granules, which increases the average diameter of starch granules [3].

Crop straw returning (SRT) significantly decreased tuber dry matter content and amylose content in both cultivars. This is consistent with the study that SRT had a significantly higher amylose concentration than straw removal in early rice [18]. In addition, SRT significantly decreased total starch and amylopectin content in Huayu-5, while it increased total starch and amylopectin content in Mira. SRT changes the soil structure, improves organic matter content and water holding capacity, and enhances the capacity of soil nutrient supply. On the one hand, returning straw to the fields increases material transportation. SRT treatment may provide more durable nutrients in the grain filling stage, which is favorable for the development of small starch granules [18]. On the other hand, SRT may cause changes in the expression and activity of key enzyme genes in starch synthesis in dryland potato tubers, thereby affecting the gelatinization properties and accumulation of synthesized starch. SRT treatment was equivalent to the role of organic fertilizer application for an increase in starch branching enzymes, and enhanced the degree of starch branching in the grains during the filling process [33]. Returning straw to the field also increased the SSS enzyme activity and SSII and SSIII gene expression levels of dryland potato, thereby changing the physicochemical properties of starch [34].

Potato intercropping with maize can reduce photosynthetically active radiation and micro-environment temperatures [35], which change the starch structure, functionality, and textural properties. In the potato/maize association, the maize plant can reduce irradiance because potato height seldom exceeds 1 m in the field against up to 3 m for maize. Plants under low-light environments decrease leaf photosynthetic rate and hinder assimilate accumulation, which leads to a reduction in the development of amyloplasts and affects the starch structure and textural properties [17]. Therefore, the starch content, amylose, amylopectin, and relative crystallinity significantly decreased under low-light conditions. Intercropping conditions changing soil temperature may be another source that provides a beneficial microclimate for potato tuber growth and affects the starch physical chemistry. Sharaiha and Battikhi [36] found that intercropped potatoes have cooler micro-environments than monocultured ones, with lower soil and air temperatures, which benefit the growth and tuber development of potatoes. The low growth temperature increased the proportion of amylopectin short chains and the molecular size of amylose. In this study, the potato monoculture treatment resulted in an increase in the medium diameter starch granule compared to that in the MIP system. Guo et al. [37] also reported that the low growth temperature decreased the average diameter of starch granules and reduced the volume-based percentage of larger-sized starch granules.

### 4.2. Effects of Crop Management Practices on the Potato Starch Physicochemical and Textural Properties

The physicochemical properties of potato starch are closely related to the starch granule size [7]. In this study, the medium diameter starch granule was positively related to the gelatinization temperatures, peak and breakdown viscosity, 1045/1022 cm^−1^ and 1022/995 cm^−1^ ratio, and adhesiveness, while negatively related to specific surface area. The changes in starch chemical compositions and molecular structures under PMF synergistically alter the gelatinization and retrogradation of potato starch. The swelling power was observed to be highest for the small-granule fractions, while the solubility was lower for these fractions than for the medium- and large-granule fractions [38]. Previous studies observed that elevated temperature increased peak viscosity, breakdown, and pasting temperature, but decreased the setback of rice flour in middle rice cropping systems. Singh et al. [39] studied starches from 46 lines of rice bean germplasm and found a positive relationship between pasting properties (peak viscosity, trough viscosity, and breakdown viscosity) and large-size granules (>30 μm). Peak viscosity, hold-through, setback, final viscosity, peak time, and pasting temperature under the PMF treatment were all lower than those under the control treatment [30].

SRT can also improve eating quality by changing starch structural, physicochemical, and textural properties [18,40]. SRT significantly increased the breakdown value and amylose content of starch in dryland potatoes, but concurrently induced notable reductions in starch peak, trough, and final viscosity [34]. SRT increased pasting temperature and decreased the peak, hot, and breakdown viscosity of milled rice [11]. In this study, the SRT increased the onset temperature (To), peak gelatinization temperature (Tp), and conclusion temperature (Tc), while decreasing the pasting time of starch. The gelatinization temperatures in this study were similar to the results in the previous reports [18]. SRT had lower To in early rice [40]. Gelatinization enthalpy was found to be highly and positively correlated with the relative crystallinity of starch, suggesting that more energy is required to break the double helix structure of starch under SRT treatment. Higher biochar application rates significantly increased the peak and breakdown viscosities and decreased the setback viscosity [11]. Textural properties of cooked potatoes were significantly affected by SR. SRT had a lower hardness or higher stickiness in early rice and late rice [40]. In this study, adhesiveness and cohesiveness were increased by SRT.

The multi-cropping system is the simultaneous cultivation of two or more crops in the same field, practiced in many regions of the world, which can enhance crop productivity and food security. Potato/maize relay intercropping is widely practiced in many regions of the world. The daily maximum photosynthetic active radiation (PAR) in this region during summer was 1494.52 μmol m^−2^ s^−1^, and the daily average PAR was 1075.56 μmol m^−2^ s^−1^ [41]. Maize was taller than potato and intercepted up to 80% of the incident radiation [42]. The light saturation of potato is approximately 400 μmol m^−2^ s^−1^ PAR [43]. Under intercropping conditions, the PAR at the canopy tops of potato plants remained below the light saturation point. Liu et al. [44] showed that low light after anthesis reduces amylose content of wheat regardless of the initial amylose content of the cultivar. Deng et al. [45] reported that low-light treatment decreased the A chain proportion of amylopectin and the amylose content in middle-season indica rice, made the crystallinity degree and 1045/1022 cm^−1^ ratio decrease, and reduced starch enthalpy change and uniformity. Liu et al. [44] found that low light inhibited starch synthesis, and the effects on amylose were greater than those of amylopectin. In this study, the MIP led to decreased dry matter and amylose content, which, in turn, can increase gelatinization temperatures, pasting viscosities, adhesiveness, and cohesiveness. The effect of the medium diameter starch granule by the MIP between two cultivars was significantly different. The MIP had a significant effect on the peak viscosity and hot viscosity of Mira, while it had no significant effect on Huayu-5. The main reason may be that Huayu-5 is a mid–early maturing potato variety, while Mira is a late maturing potato variety. With plant growth, the leaf senescence of Huayu-5 is already no longer photosynthesizing when the maize begins to shade the potato.

## 5. Conclusions

This research demonstrates the crop management practices can significantly change starch physical chemistry and the sensory characteristic of texture. Potato/maize relay intercropping increased the thermal properties, pasting viscosities, adhesiveness, and cohesiveness, with stronger effects observed in the medium–late maturing variety compared to the medium–early maturing variety. This knowledge may be of use to provide a theoretical foundation for the development that improves food quality and safeguards human health.

## Figures and Tables

**Table 1:** Dry matter, total starch, amylose, amylopectin content, and amylose/amylopectin ratio.

Variety	Treatment	Dry Matter	Total Starch	Amylose Content	Amylopectin Content	Amylose/ Amylopectin
		%	%	%	%	%
Huayu-5	A1B1C1	20.31 ± 0.16 a	78.01 ± 1.01 a	14.36 ± 0.53 a	63.65 ± 0.56 a	22.56 ± 0.71 a
	A1B1C2	18.62 ± 0.57 bc	70.96 ± 0.50 d	13.41 ± 0.46 ab	60.56 ± 0.52 b	23.29 ± 0.89 a
	A1B2C1	19.55 ± 0.48 ab	75.91 ± 1.13 ab	12.44 ± 0.44 bc	63.47 ± 0.70 a	19.59 ± 0.47 b
	A1B2C2	17.80 ± 0.21 c	71.49 ± 0.52 cd	11.59 ± 0.31 cd	59.89 ± 0.21 b	20.02 ± 0.83 b
	A2B1C1	19.06 ± 0.43 b	73.73 ± 0.73 bc	12.31 ± 0.49 cd	61.43 ± 0.29 b	20.03 ± 0.73 b
	A2B1C2	16.11 ± 0.12 d	62.10 ± 0.69 e	11.29 ± 0.12 de	50.81 ± 0.57 c	22.23 ± 0.03 a
	A2B2C1	16.04 ± 0.59 d	71.73 ± 1.42 cd	11.78 ± 0.12 cd	59.95 ± 1.37 b	19.66 ± 0.40 b
	A2B2C2	14.52 ± 0.32 e	59.88 ± 0.15 e	10.39 ± 0.37 e	49.49 ± 0.30 c	20.00 ± 0.87 b
	A	***	***	***	***	**
	B	***	***	***	**	***
	C	***	***	***	***	**
	A × B	***	ns	**	ns	**
	A × C	ns	***	ns	***	ns
	B × C	ns	ns	ns	ns	ns
	A × B × C	*	ns	ns	ns	ns
Mira	A1B1C1	23.71 ± 0.14 a	73.00 ± 0.86 c	13.91 ± 0.09 a	59.09 ± 0.89 d	23.54 ± 0.43 a
	A1B1C2	21.97 ± 0.79 bc	63.73 ± 0.43 f	12.12 ± 0.10 c	53.95 ± 0.05 f	22.47 ± 0.16 ab
	A1B2C1	22.48 ± 0.17 b	75.80 ± 0.23 b	13.31 ± 0.27 ab	62.49 ± 0.25 b	21.30 ± 0.49 bc
	A1B2C2	21.54 ± 0.31 c	66.78 ± 0.70 e	10.15 ± 0.26 de	56.63 ± 0.55 e	17.92 ± 0.41 e
	A2B1C1	21.44 ± 0.18 c	75.17 ± 1.25 bc	13.09 ± 0.22 b	62.08 ± 1.03 bc	21.08 ± 0.00 c
	A2B1C2	19.25 ± 0.27 d	64.21 ± 0.18 f	10.57 ± 0.31 d	53.63 ± 0.21 f	19.72 ± 0.64 d
	A2B2C1	21.40 ± 0.21 c	78.36 ± 1.61 a	11.74 ± 0.23 c	66.62 ± 1.38 a	17.62 ± 0.04 e
	A2B2C2	18.05 ± 0.16 e	69.61 ± 0.83 d	9.66 ± 0.33 e	59.95 ± 0.88 cd	16.12 ± 0.66 f
	A	***	**	***	***	***
	B	***	***	***	***	***
	C	***	***	***	***	***
	A × B	ns	**	ns	**	ns
	A × C	***	*	ns	**	*
	B × C	ns	ns	*	ns	**
	A × B × C	***	**	***	ns	**

Mean values from duplicate measurements with different letters in the same column are significantly different (*p* < 0.05). *, significance at *p* < 0.05; **, significance at *p* < 0.01; ***, significance at *p* < 0.001; ns, not significant at *p* > 0.05. A1: potato monoculture; A2: the potato/maize relay intercropping system. B1: without maize straw; B2: maize straw returning. C1: without mulching; C2: plastic film mulching.

**Table 2:** Effects of the crop management practices on starch granule size distribution.

Variety	Treatment	Volume Average	Specific	Medium	Volume Fraction of Different Diameter Starch Granule (%)		
		Particle Size (μm)	Surface Area (m^2^/kg)	Diameter (μm)	<30 μm	30–50 μm	>50 μm
Huayu-5	A1B1C1	50.13 ± 0.29 b	145.73 ± 2.84 ab	42.13 ± 0.51 d	17.93 ± 0.61 b	40.83 ± 0.43 b	42.83 ± 2.63 ab
	A1B1C2	48.6 ± 0.36 c	135.50 ± 0.36 d	44.67 ± 0.15 bc	17.75 ± 0.18 b	43.87 ± 0.51 a	43.66 ± 1.94 a
	A1B2C1	47.4 ± 0.57 cd	142.13 ± 0.15 bc	45.93 ± 0.15 b	21.12 ± 0.60 a	39.57 ± 0.28 b	41.56 ± 1.46 ab
	A1B2C2	56.13 ± 0.71 a	121.27 ± 0.97 e	47.63 ± 0.15 a	12.66 ± 0.46 d	36.83 ± 1.27 c	39.94 ± 3.06 b
	A2B1C1	50.38 ± 0.79 b	148.83 ± 0.95 a	41.90 ± 0.46 d	15.08 ± 0.51 c	44.1 ± 0.95 a	43.31 ± 1.39 ab
	A2B1C2	47.37 ± 0.50 cd	135.50 ± 0.56 d	43.97 ± 0.64 c	15.91 ± 0.52 c	43.43 ± 0.86 a	44.31 ± 2.42 a
	A2B2C1	46.63 ± 0.67 d	140.83 ± 1.50 c	45.73 ± 0.71 b	21.46 ± 0.54 a	44.05 ± 0.63 a	42.83 ± 3.4 ab
	A2B2C2	50.53 ± 0.15 b	122.73 ± 1.10 e	47.57 ± 0.42 a	11.43 ± 0.44 d	39.74 ± 1.78 b	40.96 ± 1.89 ab
	A	**	ns	ns	***	***	ns
	B	ns	***	***	ns	***	*
	C	*	***	***	***	**	ns
	A × B	ns	ns	ns	**	*	ns
	A × C	ns	ns	ns	ns	**	ns
	B × C	***	***	ns	***	***	ns
	A × B × C	ns	*	ns	**	ns	ns
Mira	A1B1C1	41.37 ± 0.06 c	169.23 ± 2.18 c	39.13 ± 0.06 cd	34.75 ± 0.46 b	41.45 ± 0.24 bc	23.80 ± 0.25 c
	A1B1C2	43.57 ± 0.49 a	157.20 ± 1.51 d	40.57 ± 0.31 b	28.46 ± 1.2 cd	44.13 ± 1.54 ab	27.40 ± 0.38 b
	A1B2C1	38.93 ± 0.23 e	160.90 ± 2.77 d	39.80 ± 0.20 bc	25.89 ± 1.16 de	42.25 ± 0.63 bc	32.19 ± 1.48 a
	A1B2C2	42.37 ± 0.21 b	146.37 ± 2.90 e	42.70 ± 0.50 a	24.71 ± 2.33 e	41.55 ± 2.26 bc	32.98 ± 2.11 a
	A2B1C1	36.43 ± 0.15 g	188.53 ± 0.47 a	33.67 ± 0.38 g	39.04 ± 0.94 a	39.97 ± 0.72 c	21.00 ± 0.60 cd
	A2B1C2	39.83 ± 0.3 d	167.53 ± 1.86 c	37.13 ± 0.06 e	36.02 ± 1.08 ab	41.66 ± 0.79 bc	22.32 ± 0.33 cd
	A2B2C1	37.23 ± 0.21 f	180.33 ± 0.84 b	36.20 ± 0.35 f	35.96 ± 1.64 ab	43.57 ± 0.82 ab	20.47 ± 1.08 d
	A2B2C2	40.95 ± 0.33 c	162.17 ± 0.95 d	38.40 ± 0.10 d	30.74 ± 0.54 c	46.27 ± 0.50 a	22.98 ± 0.73 cd
	A	***	ns	***	***	ns	***
	B	***	***	***	***	ns	***
	C	***	***	***	***	*	***
	A × B	***	ns	*	*	**	***
	A × C	***	ns	*	ns	ns	ns
	B × C	***	ns	ns	ns	ns	ns
	A × B × C	**	ns	***	**	*	ns

Mean values from duplicate measurements with different letters in the same column are significantly different (*p* < 0.05). *, significance at *p* < 0.05; **, significance at *p* < 0.01; ***, significance at *p* < 0.001; ns, not significant at *p* > 0.05. A1: potato monoculture; A2: the potato/maize relay intercropping system. B1: without maize straw; B2: maize straw returning. C1: without mulching; C2: plastic film mulching.

**Table 3:** Effects of the crop management practices on thermal properties.

Variety	Treatment	Peak Viscosity	Hot Viscosity	Final Viscosity	Breakdown Viscosity	Setback Viscosity
		(cP)	(cP)	(cP)	(cP)	(cP)
Huayu-5	A1B1C1	2456.67 ± 132.59 e	1226.00 ± 10.54 d	2361.00 ± 113.93 e	1230.67 ± 122.14 d	1135.00 ± 103.48 b
	A1B1C2	2872.00 ± 100.06 d	1565.67 ± 16.8 b	2657.67 ± 63.06 cd	1306.33 ± 83.76 cd	1092.00 ± 79.17 b
	A1B2C1	3166.33 ± 129.25 bc	1428.33 ± 25.32 c	2834.33 ± 61.08 bc	1738.00 ± 105.00 b	1406.00 ± 77.66 a
	A1B2C2	3745.00 ± 76.27 a	1771.33 ± 43.06 a	3032.67 ± 28.92 a	1973.67 ± 35.85 a	1261.33 ± 66.71 ab
	A2B1C1	2404.00 ± 107.53 e	1212.33 ± 13.05 d	2336.67 ± 73.24 e	1191.67 ± 103.02 d	1124.33 ± 83.74 b
	A2B1C2	3017.33 ± 115.37 cd	1542.33 ± 28.59 b	2616.33 ± 81.73 d	1475.00 ± 88.76 c	1074.00 ± 110.31 b
	A2B2C1	3304.33 ± 42.55 b	1418.33 ± 22.48 c	2905.67 ± 6.66 ab	1886.00 ± 20.66 ab	1487.33 ± 26.50 a
	A2B2C2	3777.33 ± 50.62 a	1802.33 ± 24.17 a	3074.33 ± 52.62 a	1975.00 ± 40.63 a	1272.00 ± 76.18 ab
	A	ns	ns	ns	ns	ns
	B	***	***	***	***	***
	C	***	***	***	***	**
	A × B	ns	ns	ns	ns	ns
	A × C	ns	ns	ns	ns	ns
	B × C	ns	ns	ns	ns	ns
	A × B × C	ns	ns	ns	*	ns
Mira	A1B1C1	2243.33 ± 79.43 c	1095.00 ± 15.13 e	2054.33 ± 113.54 c	1148.33 ± 70.30 cd	959.33 ± 102.75 c
	A1B1C2	2499.33 ± 80.59 bc	1125.67 ± 21.22 de	2347.33 ± 96.34 b	1373.67 ± 86.63 bc	1221.67 ± 97.03 b
	A1B2C1	2625.00 ± 97.78 b	1155.67 ± 6.66 cd	2345.67 ± 121.01 b	1469.33 ± 104.43 b	1190.00 ± 126.48 bc
	A1B2C2	2972.33 ± 80.13 a	1197.67 ± 4.93 bc	2172.33 ± 127.54 bc	1774.67 ± 84.33 a	974.67 ± 131.50 bc
	A2B1C1	1919.00 ± 34.04 d	1162.00 ± 11.53 cd	2272.67 ± 55.58 bc	757.00 ± 27.22 e	1110.67 ± 47.90 bc
	A2B1C2	2330.00 ± 106.41 c	1236.00 ± 11.27 ab	2387.00 ± 61.99 ab	1094.00 ± 105.10 d	1151.00 ± 70.77 bc
	A2B2C1	2443.33 ± 93.49 bc	1132.00 ± 23.64 de	2626.67 ± 60.87 a	1311.33 ± 112.08 bcd	1494.67 ± 47.50 a
	A2B2C2	2676.00 ± 150.37 b	1272.00 ± 19.31 a	2411.33 ± 76.46 ab	1404.00 ± 140.18 bc	1139.33 ± 70.73 bc
	A	***	***	***	***	**
	B	***	***	**	***	*
	C	***	***	ns	***	ns
	A × B	ns	***	ns	ns	*
	A × C	ns	***	ns	ns	*
	B × C	ns	**	***	ns	***
	A × B × C	ns	*	ns	ns	ns

Mean values from duplicate measurements with different letters in the same column are significantly different (*p* < 0.05). *, significance at *p* < 0.05; **, significance at *p* < 0.01; ***, significance at *p* < 0.001; ns, not significant at *p* > 0.05. A1: potato monoculture; A2: the potato/maize relay intercropping system. B1: without maize straw; B2: maize straw returning. C1: without mulching; C2: plastic film mulching.

## Data Availability

The original contributions presented in the study are included in the article. Further inquiries can be directed to the corresponding authors.
